# Hyper-Interleukin-6 Protects Against Renal Ischemic-Reperfusion Injury—A Mouse Model

**DOI:** 10.3389/fsurg.2021.605675

**Published:** 2021-05-13

**Authors:** Mohammad Zuaiter, Jonathan H. Axelrod, Galina Pizov, Ofer N. Gofrit

**Affiliations:** ^1^Department of Urology, Hadassah Hebrew University Hospital, Jerusalem, Israel; ^2^Goldyne Savad Institute of Gene Therapy, Hadassah Hebrew University Hospital, Jerusalem, Israel; ^3^Department of Pathology, Hadassah Hebrew University Hospital, Jerusalem, Israel

**Keywords:** ischemia-reperfusion injury, IL-6, nephrectomy, hyper-interleukin-6, creatinine

## Abstract

**Background:** Most of the ischemia-reperfusion injury (IR-I) occurs during reperfusion and is mediated by the immune system. In this study we determined whether immunomodulation with hyper-Interleukin-6 (a recombinant designer cytokine composed of interleukin-6 linked to its soluble receptor) is protective against IR-I in mice kidneys.

**Methods:** Hyper-Interleukin-6 (HIL-6) was administered by *in vivo* plasmid DNA transfection to 10 male mice. Twenty-four hours later, unilateral nephrectomy was done. IR-I immediately followed by closure of the remaining kidney vascular pedicle for 40 min. Seven mice transfected with non-coding control plasmid served as the control group. The functional and morphological effects of IR-I and its effect on mice longevity were explored. This was done by serial blood tests and by histopathology done upon sacrifice of the animals at post-operative day 7.

**Findings:** Mice pretreated with HIL-6 had a mean creatinine level at post-operative day 1 of 35.45 ± 4.03 μmol/l and mean Urea level was 14.18 ± 2.69 mmol/l, whereas mean creatinine was 89.33 ± 69.27 μmol/l (*P* = *0.025*), and mean urea was 38.17 ± 20.77 mmol/l *(P* = *0.0024*) in the control group. Histological changes in the control group included inflammatory infiltration, tubular damage, and architectural distortion. These were not seen in the treatment group. Seven days post-operatively the survival rate of treated mice was 100% compared to 50% in the control group (*P* = *0.015*).

**Interpretation:** In this single kidney mouse model, pretreatment with HIL-6 administration effectively protected against IR-I both morphologically and functionally. Further studies are needed to better understand the mechanism and feasibility of using this immunomodulator.

## Introduction

Renal cancer accounts for about 5% of all newly diagnosed malignancies in men and 3% in women (1). The incidence rate of renal cancer is increasing steadily since the 1970s at an annual rate of 1% (2). Most of the increase is in small stage I tumors while the incidence of stages II, III, and IV disease is decreasing (3). This change is accompanied by a steady improvement in patients longevity raising the importance of renal function preservation while caring for these patients (4). Additionally, about 25–30% of the patients with small renal masses are also affected by some degree of chronic kidney disease (CKD) even before surgery. This is due to commonly shared risk factors including older age, diabetes mellitus, and hypertension (5). Grade 3 or higher CKD is expected to develop in a third of the patients postoperatively, putting these patients at a high risk of death (24% within 5 years), mostly from cardiovascular causes, further stressing the significance of renal function preservation (6, 7).

Partial nephrectomy is the preferred surgery in most patients diagnosed with small renal mass. It provides excellent local control and minimizes the risk of CKD (8). In most cases, this surgery requires occlusion of the renal artery for some time subjecting the kidney to ischemic-reperfusion injury (IR-I). Blockage of circulation to an organ results in a burst of free radicals such as reactive oxygen species (ROS), hydrogen superoxide generated by the sudden resumption of full metabolic activity upon reperfusion, together with cytokines secreted by cells of the immune system (9, 10). Many attempts were done to decrease IR-I induced renal damage including preclamping administration of mannitol, angiotensin converting enzyme inhibitor, dopamine, and even by controlled hypotensive anesthesia. All showed no difference in renal function (11).

One of the most interesting cytokines involved in IR-I is interleukin-6 (IL-6). This cytokine acts on a limited target tissues and cell types that express its cognate, membrane-bound receptor (IL-6R), by forming a complex with it and its co-receptor, gp130, in a mechanism called *classical* signaling (12). Studies in animal models have demonstrated that for various tissues, including liver and heart, IR-I can be substantially ameliorated by either direct or indirect induction of (IL-6) signaling (13, 14). The kidney however, expresses IL-6R protein and mRNA at very low levels in comparison to the liver and does not respond to IL-6 administration alone (10). The IL-6R is also produced in a soluble-form (sIL-6R) that when complexed with IL-6 can, in a mechanism called *trans-*signaling, initiate signaling in gp130 expressing cells, including in cells that do not express membrane-bound IL-6R (15). Therefore, IL-6 *trans*-signaling acts as an agonist on cell types that, although expressing gp130, would not inherently respond to IL-6 alone (16, 17). Indeed, as we have shown previously, IL-6 *trans-*signaling by the recombinant IL-6/sIL-6R fusion protein, Hyper-IL-6 (HIL-6) (18) can strongly upregulate STAT3 phosphorylation in the kidney and largely prevent ROS-mediated acute kidney injury (AKI) (10, 19). The potential of IL-6 *trans*-signaling to prevent IR-I induced AKI in the context of partial nephrectomy is unknown.

We have utilized a mouse model of solitary kidney IR-I to study the potential of HIL-6 in preventing IR-I induced renal failure.

## Materials and Methods

Seventeen BALB/c male mice, seven to 8-week old, with a mean weight of 23 g were purchased from Harlan Laboratories (Jerusalem, Israel). Mice were maintained in an SPF animal facility with a temperature of ~23°C in a 12-h light-dark cycle and received sterile commercial rodent chow and water ad-libitum. Maintenance of mice and all experimental procedures and were performed in accordance with the Institutional Animal Care and Use Committee approved animal treatment protocols (license number OPRR-A01-5011).

Surgeries were done under general anesthesia, and with a retro-peritoneal surgical access to both kidneys. For anesthesia we used Ketamine (1.25 microl/gr), injected intra-peritoneally, and Xylazine with a concentration of (23 mg/ml). After anesthetizing the mice, bilateral retroperitoneal incisions were made using a scalpel. Right sided nephrectomy was performed first and the renal pedicle was closed with a vicryl suture. Shortly afterwards, the left renal pedicle was clamped for 40 min in order to induce Ischemia reperfusion injury to the left kidney essentially as described by Michels et al. (20) and Hesketh et al. (21). Skin incisions were closed with intermittent absorbable vicryl sutures 0/2. We followed the mice for 7 days post-operatively with serial blood tests taken from the tail vein. Blood samples were obtained on post-operative days 1, 3, and 7 for determination of creatinine and urea levels. Mice were sacrificed at post-operative day 7. Kidney biopsies were obtained, formaldehyde fixed in for 24 h, paraffin embedded and H&E stained for histopathological evaluation.

**Figure 1:**
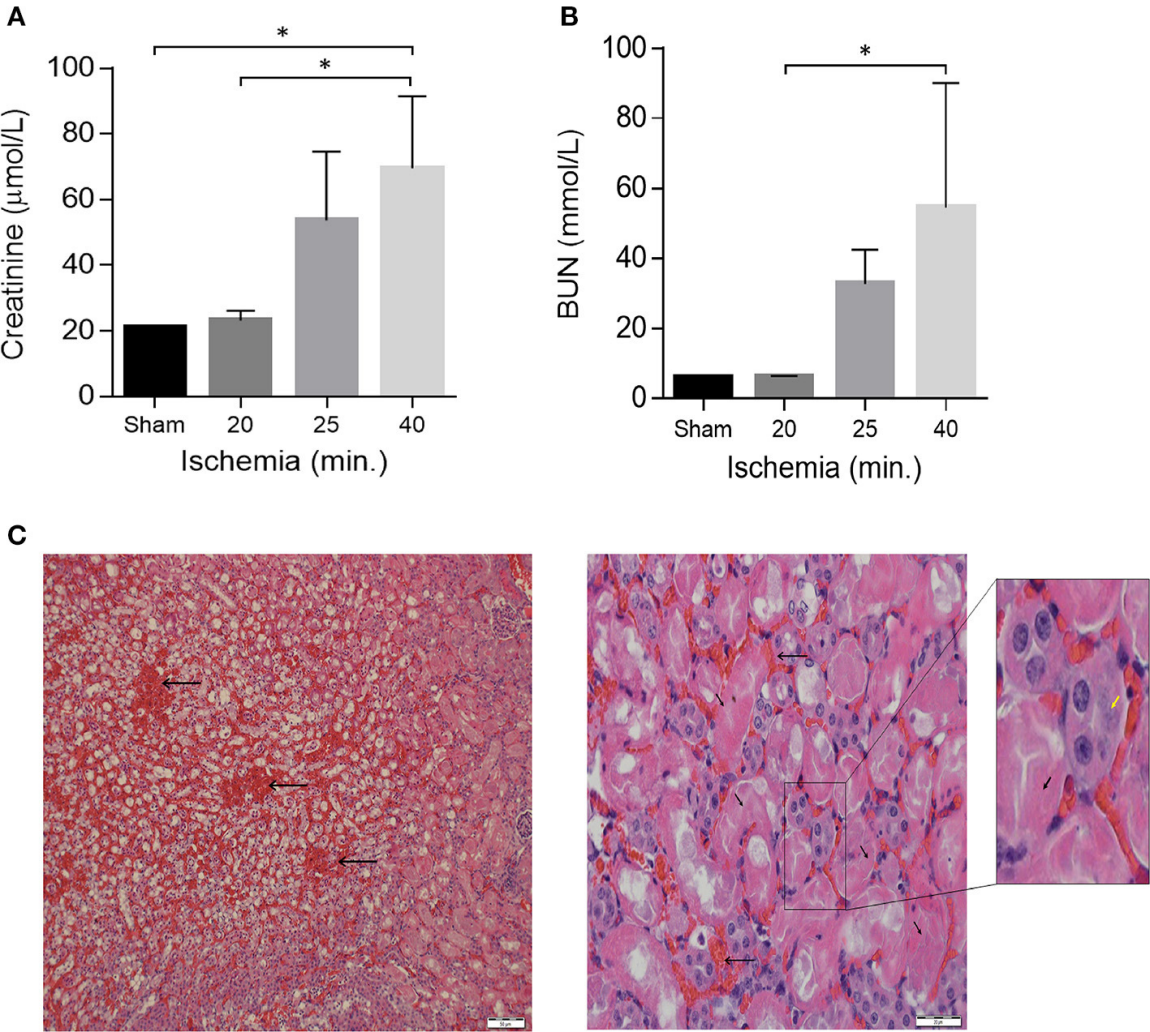
Acute kidney Injury in a model of unilateral nephrectomy with IR-I of the contralateral kidney. **(A)** Serum creatinine and **(B)** serum urea levels 24 h post-operation. Sham (*n* = 2), 16–20 min ischemia (*n* = 7); 21–25 min ischemia (*n* = 6); 40 min. ischemia (*n* = 4). Data are presented as means ± SD. *P < 0.05 by Kruskal-Wallis one-way ANOVA test with Dunn's *post-hoc* multiple comparison test. **(C)** H&E staining of kidney sections 1 day after 40 min of ischemia showing extensive tubular necrosis (thick arrows), ghost cells (yellow arrowhead), dilation of the tubular lumina filled with proteinaceous material and congestion (thin arrows). Scale bars, 50 μM (x40) and 20 μM (x400).

Mice were assigned into two trials. Trial A (*n* = 20) was done to determine the optimal ischemia duration for IR-I (calibration of the system). Mice were operated in different ischemia times; 0, 16, 18, 20, 22, 24, 26, 30, 35, 40, and 60 min. Two mice out of the 20 underwent right nephrectomy without I-RI. Three mice died during this experiment on ischemia times 20, 24, and 60 min. On blood tests post-operative day 1, creatinine levels started to deteriorate after 22 min of ischemia (44 μmol/l) and urea levels started to rise after 24 min of ischemia (42 mmol/l); (Figure 1). Based on the mean creatinine and urea levels, 1 day after performing ischemia-reperfusion + right sided nephrectomy, we decided that the optimal ischemia time to carry out the second trial was 40 min of ischemia-reperfusion. Worth noting that the mean creatinine level after one sided nephrectomy without contralateral ischemia was (21 μmol/l) and the mean urea level was (6.1 mmol/l).

In Trial B (*n* = 17), IR-I was done 24 h after HIL-6 injection. Mice were subdivided into two groups: experimental group (*n* = 10) and control group (*n* = 7). The immunomodulatory agent “HIL-6” was injected into the experimental group (*n* = 10) and placebo “Plasmid” was injected to the control group (*n* = 7), through the tail vein. Right nephrectomy plus 40 min of ischemia-reperfusion injury to the left kidney was carried out 24 h later in both groups.

HIL-6 injection was performed by hydrodynamics-based *in vivo* plasmid DNA transfection 24 h before surgery as previously described using the expression plasmid with phAAT-HIL-6 (2.5 μg) and with pGEM-7 (17.5 μg) (10). Control mice were transfected with pGEM-7 (20 μg) alone. Serum creatinine and urea were obtained at post-operative days one, three and seven. Renal histopathology was also obtained and compared after scarifying mice. In addition, overall survival was compared between both groups.

## Statistical Analysis

Data were evaluated for significance by two-tailed Mann-Whitney test, one-way ANOVA, or log-rank test, as indicated, with *P* ≤ 0.05 considered significant. Calculations were performed using GraphPad Prism 6.02 software (Graph-Pad Software, Inc., San Diego, CA).

## Results

From the Trial A (*n* = 20) we found out that by increasing ischemia times from 16 to 40 min the mice showed proportionally increasing renal failure manifested by elevated creatinine and BUN levels on post-operative day 1 (Figures 1A,B). Histological examination of kidney sections from mice sacrificed on post-operative day-1 confirmed the presence of acute renal injury, particularly following 40 min of IR-I, revealing extensive tubular necrosis, ghost cells, dilation of the tubular lumina filled with proteinaceous material, and congestion (Figure 1C). After establishment of the model, a 40 min ischemia period was chosen for the next step of the study.

**Figure 2:**
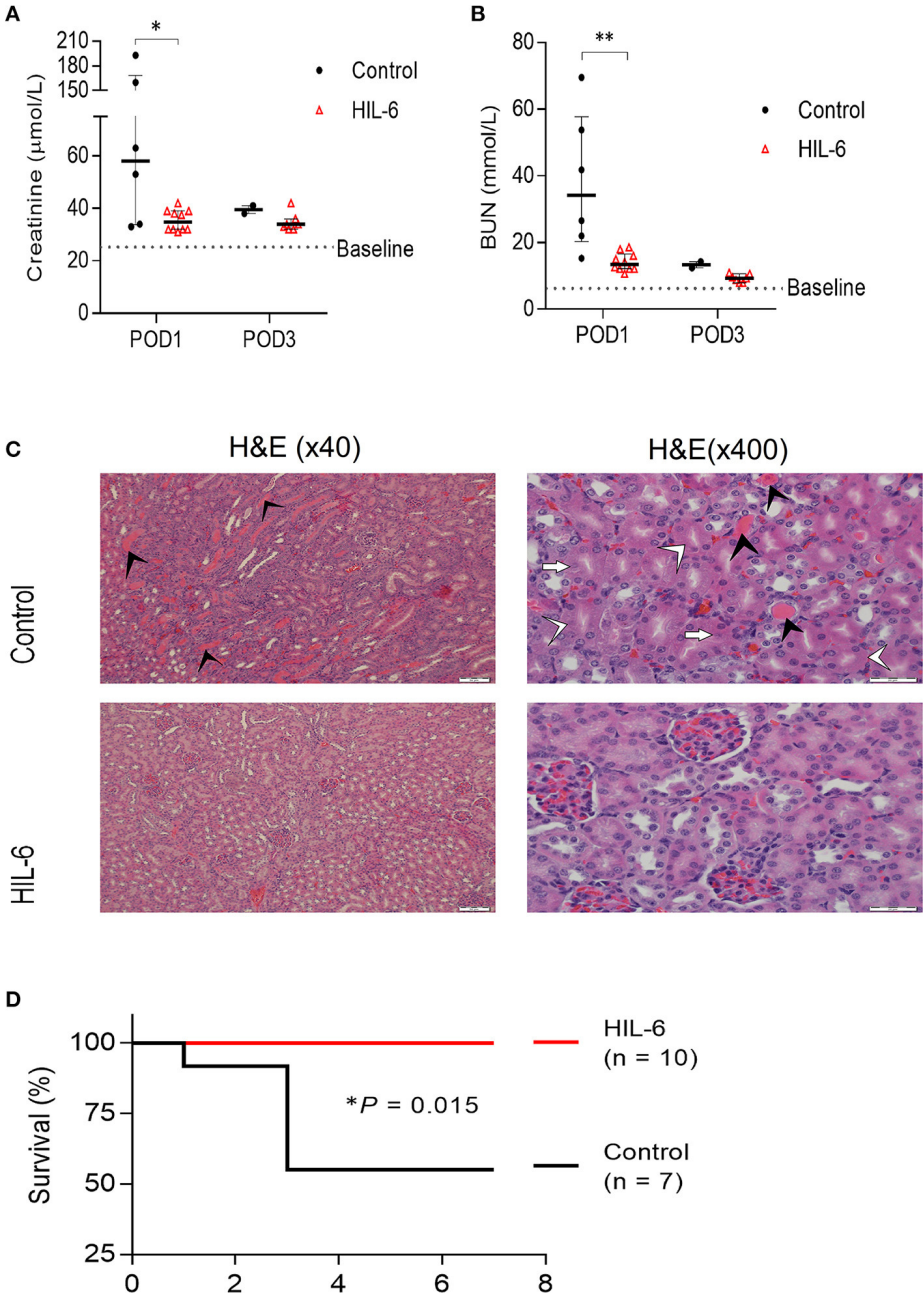
HIL-6 prevents acute kidney Injury in a model of unilateral nephrectomy and immediate 40 min ischemia-of the contralateral kidney. Serum creatinine **(A)** and urea **(B)** assessed at post-operative days one and three. Data are presented as medians (bar) ± IQR and individual mice. *P < 0.05 and **P < 0.01 by Mann-Whitney test. **(C)** Representative H&E stained kidney thin sections from mice on post-operative day 3 showing extensive tubular necrosis (white arrowheads), ghost cells (white arrows), dilation of the tubular lumina filled with proteinaceous material (black arrowheads), and congestion in kidneys of control treated mice, but near-normal kidney morphology in HIL-6 treated mice. Scale bars, 50 μM (x40) and 20 μM (x400). **(D)** Kaplan-Meir plot of survival of HIL-6 and control treated mice following unilateral nephrectomy with ischemia. *P = 0.015 by Log rank test.

In Trial B (*n* = 17), mice were either pretreated with either HIL-6 protein (*n* = 10), administrated via transient high-pressure transfection of a pDNA expression vector, or with control pDNA (*n* = 7) (10). Administration of HIL-6 via pDNA transfection this way transiently produces serum HIL-6 levels reaching approximately 5 ng/ml during the first 2 days post-transfection (10). Biochemical analyses of blood serum showed that while control mice developed substantial kidney injury (*P* = *0.025* and *P* = *0.0024* for creatinine and urea, respectively), HIL-6 treated mice displayed strikingly small impairment of renal function that was not substantially different from the baseline levels (Figures 2A,B); the mean creatinine level in the experimental group was 35.45 ± 4.0 μmol/l and mean urea level was 14.2 ± 2.7 mmol/l, whereas mean creatinine was 89.3 ± 69.8 μmol/l (*P* = *0.025*), and mean urea was 38.17 ± 20.77 mmol/l (*P* = *0.0024*) in the control group on post-operative day 1. Blood tests taken 3 days after surgery showed no statistically significant differences between creatinine levels in both groups; the mean creatinine level in the control group (*n* = 2) was 39.50 ± 2.12 μmol/l and the mean creatinine level was 34.7 ± 3.5 μmol/l (*P*= *0.194*) in the HIL-6 group (*n* = 7). On the other hand, mean urea levels were statistically significant; mean urea level was 13.35 ± 1.34 mmol/l in the control group and 9.3 ± 1.2 in the HIL-6 group (*P* = *0.004*). Although statistically insignificant, creatinine, and urea levels continued to improve 7 days after surgery.

Histopathological analysis of kidney sections from mice sacrificed on post-operative day 3 revealed extensive areas with signs of acute tubular injury in the control group while the HIL-6 treated mice no signs of acute renal damage observed (Figure 2C). Importantly, while all HIL-6 treated mice survived the first week post-operation, mortality levels in the control-treated mice was 50% (*P* = *0.015*) (Figure 2D). Renal functions returned to near-normal levels in all mice that did not succumb within 3 days post-operation in both groups (Figures 1A,B).

## Discussion

The pathophysiology of IR-I in kidneys is complex and not completely understood. Furthermore, there is still no consensus in literature regarding the optimal ischemia time duration to perform the experimental ischemia reperfusion injury.

In our experimental model we proved that immunotherapy with HIL-6 is protective against (IR-I) on the short run. We found out that 40 min ischemia is the optimal time for performing the experiment. The most striking difference in blood tests results was noticed 1 day after surgery, demonstrating that IR-I impact is greatest in the first 24 h post operatively. On the other hand, at about 7 days after the surgery, blood tests almost came back to normal in both groups, and this questions the presence of a permanent injurious effect secondary to (IR-I). However, it is still unknown whether the sequalae of (IR-I) is reversible or not.

In this model it was very difficult for mice to recover from anesthesia. The mice had a single functioning kidney that was struggling to regain function after a certain period of ischemia and secondary ischemia reperfusion injury. As a result, blood levels of Ketamine and Xylazine became in a state of “apparent overdose” by the end of surgery and thus a longer recovery time. A trial of “staged anesthesia”; (i.e., diving the anesthetic dose into multiple smaller ones), resulted in mice awakenings during surgery. Therefore, any mouse which didn't recover from surgery because of overdose was excluded from the experiment. Consequently, we recommend giving inhalational anesthesia in an ischemia-reperfusion model. We also recommend soaking the mice food with water in the first 2 days after surgery.

The risk of developing grade 3 or higher CKD after partial nephrectomy reaches 1/3 (2). This is a strong driving force for continuous research in protective agents against IR-I. Kidney preservation is important in any patients but it takes an even higher priority in patients with a single kidney, either congenital or acquired, in patients with CKD, or in kidney transplant patients.

Since the immune system is responsible for most of the IR-I induced damage immunomodulation seems like a reasonable approach (9, 22). Here, using a single kidney mouse model, we have explored the efficacy of recombinant IL-6/sIL-6R fusion protein (HIL-6) in preventing IR-I induced AKI. Our findings demonstrate a striking ability of pretreatment with HIL-6 to eliminate IR-I. Pretreated mice had a minimal change in kidney function (creatinine and urea) or morphology and they lived longer (post-operative survival rate of 100% compared to 50% in the control group, *P* = 0.015).

The mechanism of the protective effect of HIL-6-mediated on ischemia-reperfusion injury has yet to be determined. However, our previous studies have shown that HIL-6 treatment strongly induces upregulation of oxidative stress response genes in the kidney, including heme oxygenase-1 (*HO-1*) and apurinic endonuclease/redox effector factor (10), the net effect of which strongly reduces the ROS-mediated injury potential (10). Since IR-I is largely an oxidative stress-mediated, we speculate that similar molecular mechanisms were responsible for kidney preservation in this model (9, 23).

Of note, it is of paramount importance to check after clamping the renal blood vessels that the kidney color is homogenously blackish, otherwise the renal clamping is not ideal. Such cases were excluded from the study.

This study has several limitations:

- In our model, all surviving mice, including control group animals, appeared to return to normal renal function levels within 7 days post IR-I, without notable long-term effects. Thus, it is not clear whether our model recapitulates the long-term sequelae of kidney injury commonly found in human patients, nor the benefit of HIL-6 pretreatment in preventing these chronic outcomes.
- Kidney function and morphology were monitored, but other biomarkers of IR-I like NGAL (neutrophilgelatinase-associated lipocalcin), NAG (N-acetyl-beta-D-glucosaminidase), or KIN-1 (kidney injury molecule-1) were not studied (11). Furthermore, the proposed protective mechanism of HIL-6 is based on its protective effect against HgCl2-induced AKI (10).
- Neither serum levels of HIL-6 nor any other event downstream to the transfection was measured. Verification of mechanisms and experiments in larger animals must be carried out before clinical experiments.

## Conclusion

In this single kidney mouse model, we showed that pretreatment with HIL-6 robustly prevents IR-I induced renal injury and increases animal survival. Since IR-I is an expected event in the clinical scenario of partial nephrectomy, protective pretreatment with HIL-6 can be envisioned if further experiments will confirm these findings.

## Data Availability Statement

The original contributions presented in the study are included in the article/supplementary materials, further inquiries can be directed to the corresponding author/s.

## Ethics Statement

The animal study was reviewed and approved by Hebrew University Jerusalem.

## Author Contributions

OG and JA conceived the study. MZ and JA designed and conducted the experiments and acquired data. OG supervised the study. MZ, GP, and JA analyzed data. MZ and JA assembled the data and wrote the manuscript. All authors discussed the results and edited the manuscript.

## Conflict of Interest

The authors declare that the research was conducted in the absence of any commercial or financial relationships that could be construed as a potential conflict of interest.
